# A High Dynamic Range and Fast Response Logarithmic Amplifier Employing Slope-Adjustment and Power-Down Mode

**DOI:** 10.3390/mi16070741

**Published:** 2025-06-25

**Authors:** Yanhu Wang, Rui Teng, Yuanjie Zhou, Mengchen Lu, Wei Ruan, Jiapeng Li

**Affiliations:** 1 Institute of Semiconductors, Chinese Academy of Sciences, Beijing 100083, China; wangyh@semi.ac.cn (Y.W.); ruanw@semi.ac.cn (W.R.); lijp@semi.ac.cn (J.L.); 2 China Electronics Technology Group Corporation 24th Research Institute, Chongqing 400060, China; zhouyuanjie2001@sohu.com (Y.Z.); lumengchen@cetcact.com (M.L.)

**Keywords:** logarithmic amplifier, limiting amplifier, log slope, power-down, response time

## Abstract

Based on the GSMC 180 nm SiGe BiCMOS process, a parallel-summation logarithmic amplifier is presented in this paper. The logarithmic amplifier adopts a cascaded structure of nine-stage fully-differential limiting amplifiers (LA) to achieve high dynamic range. The ten-stage rectifier completes the conversion of amplified voltage to a logarithmic current signal. A log slope adjuster is proposed. It can provide slopes of 17–30 mV/dB by configuring an off-chip resistor to meet the detection requirements of different input power. Meanwhile, a power-down control unit is designed to reduce the power consumption to only 162 μW in standby mode. The post-simulation results show that under 5 V power supply voltage, the dynamic range exceeds 80 dB and the 3 dB bandwidth is 20 MHz–4 GHz. It also has a fast response time of 42 ns with a power consumption of 109 mW in normal operation mode.

## 1. Introduction

In high-precision electromagnetic measurement systems such as radar, optical receiver and field strength meters, the input signal power is extremely weak. In order to achieve high-precision signal amplification within a wide dynamic range, logarithmic amplifiers have replaced automatic gain control loops (AGC) as a more effective solution in recent years [1,2,3,4]. Early logarithmic amplifiers have mostly adopted segmented detection architectures, which rely on multi-stage diode detectors in series to achieve signal compression. These circuits have a simple structure and high reliability, but they have defects such as large temperature drift and complex calibration. In addition, due to the disadvantages of limited dynamic range, large area, and insufficient compatibility with integrated circuit technology, further development cannot be achieved [5,6,7,8].

With the advancement of the CMOS and SiGe processes, the logarithmic amplifier has also developed rapidly. Its technological evolution mainly revolves around three core challenges: dynamic range expansion, temperature stability improvement, and bandwidth breakthrough. Lakshminarayanan et al. proposed an RF power detector that achieves a dynamic range of 52 dB with bandwidths of 100 MHz–4 GHz and logarithmic linearity of ±1 dB. It realized a good compromise between logarithmic accuracy and dynamic range [9]. Yong et al. presented a high dynamic range logarithmic amplifier using four channel amplification and current summation techniques with a dynamic range of 70 dB and power consumption of only 19 mW. However, its bandwidth is only 1 MHz, which makes it unsuitable for RF systems and only suitable for low-frequency signal analysis [10]. Ahuja et al. presented an ultra-low power CMOS logarithmic amplifier for low-frequency IoT sensing. This circuit has a dynamic range of 50 dB with a power supply of 1.5 V, while consuming only 14.63 μW. But its bandwidth is limited to 10 Hz–40 kHz. Hence, it is mainly used in physiological signal detection systems, and cannot adapt to the frequency response requirements of field strength meters [11]. Wongnamkam et al. designed a fully-differential logarithmic amplifier for 2.4 GHz ISM applications, which realized a voltage gain of 43 dB under a single 2.5 V power supply. However, it consumes a static current of 73.5 mA that results in power consumption of up to 183.75 mW. Hence, it has significant limitations in its energy efficiency [12]. Chuang et al. proposed a logarithmic amplifier using InP InGaAs double heterojunction technology, with a bandwidth of 22 GHz. However, its power consumption is as high as 650 mW [13].

In response to the collaborative design challenges of dynamic range, bandwidth, and energy efficiency, this paper proposes a logarithmic amplifier that integrates power-down mode and log slope adjuster. By implementing a dynamic power management strategy, the static power consumption can be reduced to 162 μW. The logarithmic amplifier is implemented based on a parallel-summation structure of 9-stage limiting amplifiers with dynamic range of over 80 dB and 3 dB bandwidth of 20 MHz–4 GHz. At the same time, to meet the detection requirements of different input power, a logarithmic slope adjustment scheme is proposed. By configuring off-chip resistors, the slope can be effectively adjusted to achieve signal detection over a large input range.

## 2. Circuit Architecture and Design

**Figure 1:**
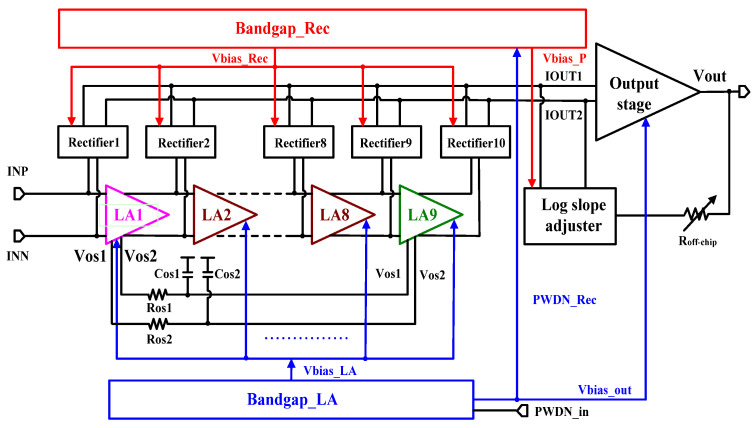
Architecture of proposed logarithmic amplifier.

The logarithmic amplifier adopts a parallel-summation structure. As shown in Figure 1, it consists of nine-stage fully-differential limiting amplifiers (LA), ten-stage full-wave rectifiers, bandgap, log slope adjuster and output stage.

Nine cascade limiting amplifiers are used to achieve signal gain expansion, and each amplifier output is equipped with a rectifier. The input dynamic range is determined by the gain and the number of cascaded logarithmic amplifier stages. The more stages there are, the larger the dynamic range which can be obtained [14]. Each limiting amplifier provides 10 dB gain and expands the input dynamic range through step-by-step gain compression. The voltage output by each limiting amplifier is input into the rectifier. The rectifier converts the output of each stage into current and sums them up at the input of output stage. The summed current signal is finally converted into a single-ended voltage through the output stage for output. The log slope adjuster introduces an off-chip adjustable resistor, combined with an internal bias adjustment circuit, to dynamically control the weight of the superimposed current, ultimately achieving adjustable slope. Two bandgaps provide bias to limiting amplifiers and rectifiers, respectively, which can avoid crosstalk between the two. The Bandgap_LA is controlled by the external power-down signal PWDN_in, which outputs signal PWDN_Rec to control the Bandgap_Rec and itself to be in normal operation or low-power standby mode, respectively. Cascaded limiting amplifiers can effectively expand the output dynamic range. However, due to the inter-stage DC coupling, when the common-mode voltage drifts, it is easy to cause oscillation [15]. Therefore, this design adds an offset correction circuit composed of Cos1/Cos2 and Ros1/Ros2. Capacitors Cos1 and Cos2, along with resistors Ros1 and Ros2, form a low-pass filter to filter out high-frequency components in feedback signals. The DC component of the voltage in the offset is fed back to the first limiting amplifier, thereby reducing the inter-stage offset caused by DC coupling.

**Figure 2:**
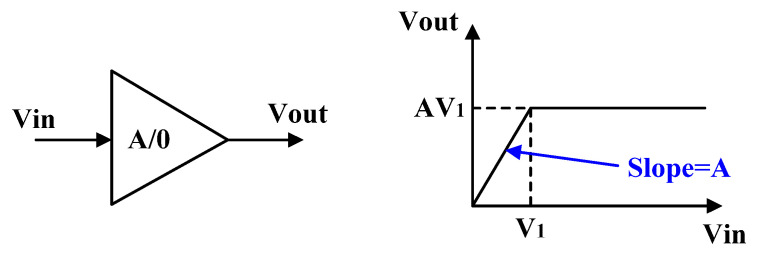
The basis of A/0 limiting amplifier.

The first to ninth limiting amplifiers use an A/0 structure, as shown in Figure 2. The input is Vin and the output is Vout. When Vin is small, Vout is amplified by A times. While Vin can reach or become larger than V_1_, Vout is limited to the maximum value of AV_1_.

When the input V_in_ is less than V_1_, the limiting amplifier exhibits a linear amplification trend with an amplification factor of A. When Vin is greater than V_1_, the amplifier enters a limiting state and outputs a fixed value [16,17]. The voltage transfer characteristic (VTC) of the A/0 limiting amplifier can be obtained as

(1) $$Vout=\left\{\begin{array}{l} AV_{in}\;0<V_{in}<V_{1}\\ AV_{1}\;V_{in}>V_{1} \end{array}\right.$$

**Figure 3:**
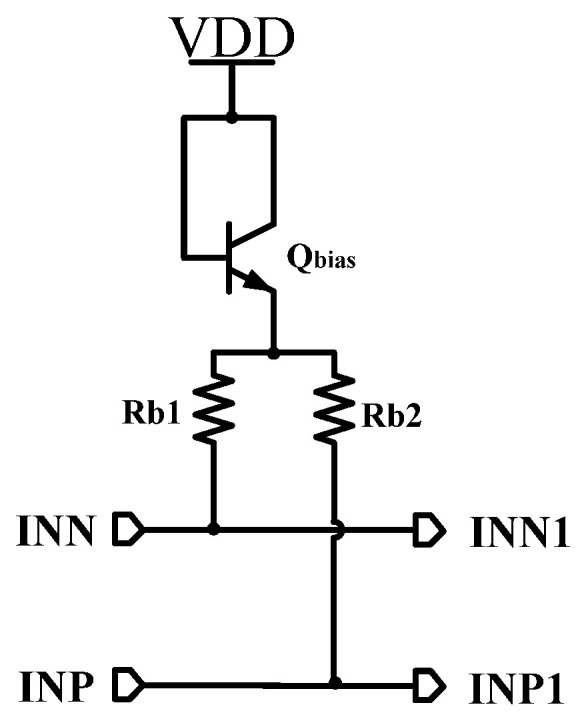
Input common-mode bias circuit.

Figure 3 shows the bias circuit of the first limiting amplifier that is mainly composed of the bias transistor Qbias and resistors Rb1 and Rb2. To simplify the design, the bias is directly generated by connecting Qbias and resistors Rb1/Rb2 in series from the power supply. The main purpose of Qbias is to reduce the temperature coefficient of the bias and obtain a more stable input common-mode bias. The common-mode operating voltage is set at 3.9 V.

**Figure 4:**
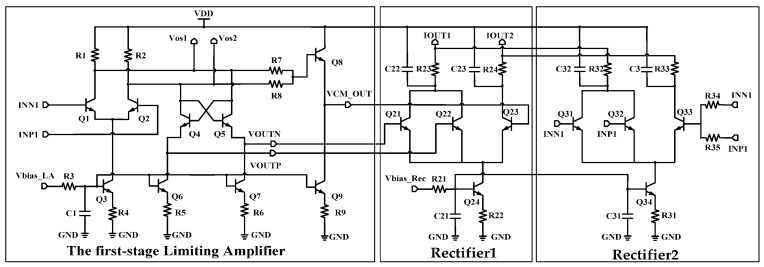
Limiting amplifier #1 and rectifiers.

Figure 4 shows the first limiting amplifier. This circuit adopts a single-stage common-emitter structure. The tail current source transistor Q3 is biased by the bias Vbias_LA output from the Bandgap_LA, and the parallel capacitor C1 serves as a decoupling element to filter out high-frequency noise. The collector current of Q3 is determined by the bias network composed of R3 and R4. This current directly constrains the transconductance of Q1 and Q2, which determines the log amplifier’s bandwidth and gain. In order to further improve gain, a cross-coupled resistor composed of Q4 and Q5 is introduced. Because the output resistance is −2R_out_, it effectively increases the voltage gain by increasing the equivalent output impedance. Transistors Q8 and Q9 form a common-mode voltage extractor, which is used to extract the common-mode output from the limiting amplifier to the rectifier. Because the gain of the first limiting amplifier is 10 dB (3.16), the output Vout is:

(2) VOUTN−VOUTP=3.16·(INN1−INP1)

The first limiting amplifier embeds two rectifiers, which respectively detect the input/output of the limiting amplifier and generate differential output currents that are logarithmically related to them. The rectifier adopts a three-port common-emitter structure, in which the differential and common-mode input are connected to rectifier2, while the differential-mode output of the first limiting amplifier and its common-mode output are connected to rectifier1 [18]. The bias of the rectifier is provided by the Bandgap_Rec, and the stability of its static operating current directly determines the logarithmic conversion accuracy. This design effectively expands the dynamic response range by simultaneously feeding differential voltage components into the multi-stage rectifier. Its working principle is that the multi-stage structure can process signals of different power in segments, while maintaining linearity and expanding the detection range [19].

In rectifier1, the output common-mode voltage of the limiting amplifier is V_cm_, the output differential-mode voltage is V_id_, and I_c21_, I_c22_, and I_c23_ are the collector currents of Q21, Q22, and Q23, respectively. The mathematical proof is as below:

(3) $$V_{cm}+\frac{V_{id}}{2}-V_{be21}=V_{cm}-V_{be23}$$

(4) $$V_{cm}-\frac{V_{id}}{2}-V_{be22}=V_{cm}-V_{be23}$$

And $I_{c}=I_{s}\cdot e^{\frac{V_{be}}{V_{T}}}$, so

(5) $$V_{T}\ln\frac{I_{c21}}{I_{s21}}-V_{T}\ln\frac{I_{c23}}{I_{s23}}=\frac{V_{id}}{2}$$

(6) $$V_{T}\ln\frac{I_{c23}}{I_{s23}}-V_{T}\ln\frac{I_{c22}}{I_{s22}}=\frac{V_{id}}{2}$$

Choose Q23 emitter area to be twice the emitter area of Q21 and Q22,

(7) $$I_{s23}=2I_{s22}=2I_{s21}$$

And

(8) $$I_{c21}+I_{c22}+I_{c23}=I_{c24}$$

combine (5), (7) and (6), (8), respectively.

(9) $$\frac{I_{c21}}{I_{c23}}=\frac{1}{2}e^{\frac{V_{id}}{2V_{T}}}$$

(10) $$\frac{I_{c23}}{I_{c22}}=2e^{\frac{V_{id}}{2V_{T}}}$$

Combine (8), (9), (10), so

(11) $$I_{c21}=\frac{1}{2}\cdot \frac{I_{c24}\cdot e^{\frac{V_{id}}{2V_{T}}}}{1+\frac{1}{2}(e^{\frac{V_{id}}{2V_{T}}}+e^{-\frac{V_{id}}{2V_{T}}})}$$

(12) $$I_{c22}=\frac{1}{2}\cdot \frac{I_{c24}\cdot e^{-\frac{V_{id}}{2V_{T}}}}{1+\frac{1}{2}(e^{\frac{V_{id}}{2V_{T}}}+e^{-\frac{V_{id}}{2V_{T}}})}$$

(13) $$I_{c23}=\frac{I_{c24}}{1+\frac{1}{2}(e^{\frac{V_{id}}{2V_{T}}}+e^{-\frac{V_{id}}{2V_{T}}})}$$

Then the output current of the rectifier is

(14) $$\Delta I=IOUT1-IOUT2=I_{c21}+I_{c22}-I_{c23}=I_{c24}\cdot \tanh^{2}(\frac{VOUTN-VOUTP}{4V_{T}})$$

**Figure 5:**
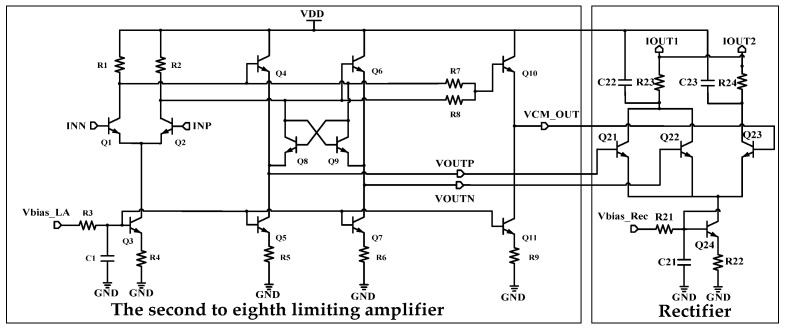
Limiting amplifiers from #2 to #8 and rectifier.

The intermediate seven limiting amplifiers adopt a structure similar to the first one, as shown in Figure 5. An emitter follower is added between the main limiting amplifier and the common-mode extractor as a buffer to isolate inter-stage interference. The input of the emitter follower is coupled to the output of the main limiting amplifier, and the common-mode component of buffer output is made to be consistent with the common-mode of the main amplifier by adjusting the Vce of Q4 and Q6. The rectifier synchronously extracts the common-mode voltage and differential output by the limiting amplifier to generate output current that is logarithmic to the input voltage.

**Figure 6:**
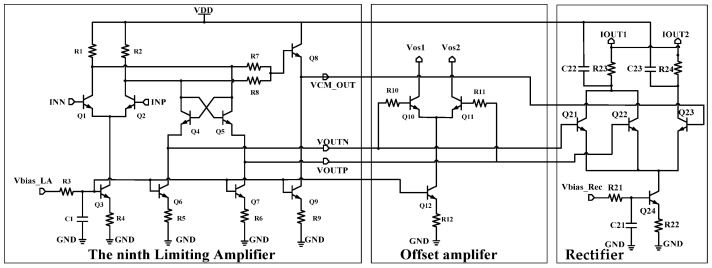
Limiting amplifier #9, offset amplifier, and rectifier.

The ninth limiting amplifier is basically the same as that of the previous stage, as shown in Figure 6. This limiting amplifier adds an offset amplifier to extract the output offset of the nine-stage limiting amplifiers, which is fed back to the first limiting amplifier in the form of a current to reduce the inter-stage offset [20,21].

**Figure 7:**
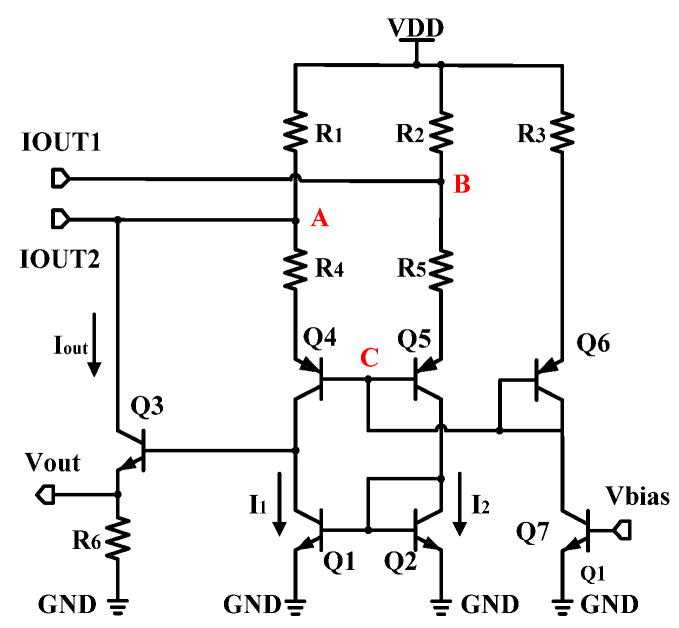
Output stage.

As shown in Figure 7, the output stage is a current mirror that converts differential input current to single-ended output voltage. By neglecting the base current and the Early effect, for node A and node B, respectively,

(15) $$\frac{V_{DD}-V_{A}}{R_{1}}+IOUT2=Iout+I_{1}$$

(16) $$\frac{V_{DD}-V_{B}}{R_{2}}+IOUT1=I_{2}$$

By setting transistors Q1 and Q2 to be exactly the same, with resistors R1 = R2, and combining (15) and (16)

(17) $$\frac{V_{B}-V_{A}}{R_{1}}=Iout+IOUT1-IOUT2$$

For the voltage of node C

(18) $$V_{A}-I_{1}\cdot R_{4}+V_{be4}=V_{B}-I_{2}\cdot R_{5}+V_{be5}\;\Rightarrow \;V_{A}=V_{B}$$

Put (18) into (17), then

(19) Iout=IOUT2−IOUT1

Finally, it can be obtained

(20) $$Vout=Iout\cdot R_{6}=(IOUT2-IOUT1)\cdot R_{6}$$

It can be seen from (20) that the difference in output current of rectifier is converted into a single-ended output voltage. The value of resistor R6 needs to be carefully designed. On one hand, it is necessary to ensure that the output voltage has sufficient amplification gain; and on the other hand, it is necessary to ensure that I1 is small and will not generate excessive power consumption in the output stage.

**Figure 8:**
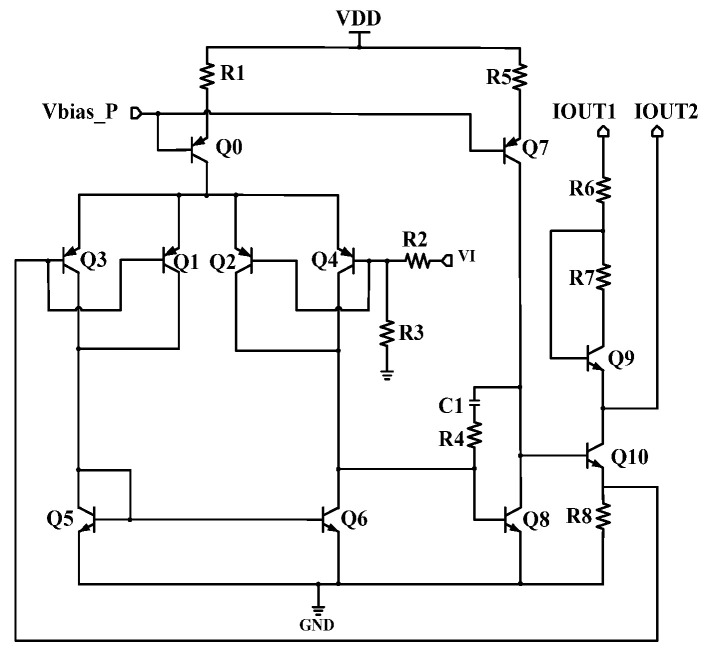
The log slope adjuster.

The log slope adjuster is shown in Figure 8, which is a two-stage amplifier and constructs a feedback loop with the output stage through an external adjustable resistor R_off-chip_. The output current of rectifiers has inherent deviation, and if directly fed into the output stage, it will cause saturation. The log slope adjuster adjusts the base voltage of Q4. Hence, the base voltage of Q10 varies, thereby controlling the difference between the output currents IOUT1 and IOUT2. When R_off-chip_ increases, V_be,Q4_ decreases. Then I_c,Q4_ and the gain of amplifier increases, which improves output voltage. Therefore, V_be,Q10_ rises to increase the output current IOUT1/IOUT2 and the log slope. However, if the R_off-chip_ decreases, the amplifier gain and output voltage also reduce. Therefore, V_be,Q10_ and the output current IOUT1/IOUT2 decrease, which finally reduces the log slope. This scheme dynamically injects the compensation current into the input of the output stage to calibrate the logarithmic transfer function. This mechanism achieves an adjustable log slope while avoiding output stage saturation.

**Figure 9:**
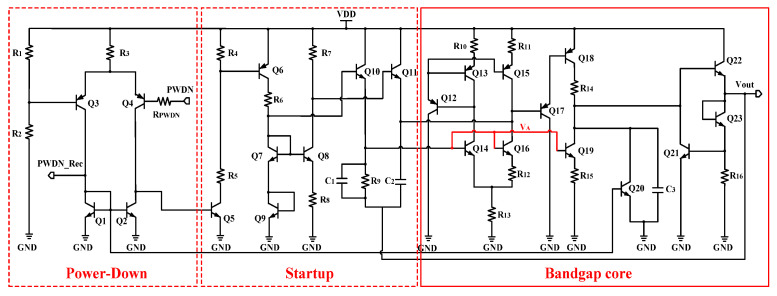
Bandgap_LA.

In Figure 9, the power-down circuit is actually a comparator. Because R1 = R2, it compares the input PWDN with VDD/2. If PWDN is high, then the base voltage of transistor Q5 becomes low. Hence, the bias current will be nullified and the Bandgap_LA will be turned off. At the same time, the output PWDN_Rec also becomes low, and the Bandgap_Rec circuit is also turned off. At this point, the overall logarithmic amplifier enters low-power standby mode. When PWDN is low, the Startup circuit works normally and starts the Bandgap core. During the power-on process, capacitor C2 is charged to remove the Bandgap from the dead zone, ensuring that the circuit can start normally. Because

(21) $$I_{R_{12}}\cdot R_{12}=\Delta V_{be}=V_{be14}-V_{be16}=V_{T}\cdot \ln n$$

$\Delta V_{be}$ has a positive temperature coefficient, and

(22) $$V_{A}=\Delta V_{be}+V_{be,Q16}=V_{T}\cdot \ln n+V_{be,Q16}$$

Since $V_{be,Q16}$ has a negative temperature coefficient, $V_{A}$ with a zero temperature coefficient is achieved to bias Q19. In addition, the currents of R_15_ and R_16_ also have a positive temperature coefficient. At the output node, due to the negative temperature dependence of $V_{be,22}$, it can be obtained

(23) $$Vout=I_{R_{16}}\cdot R_{16}+V_{be23}$$

We thus obtained the output Vout with zero temperature coefficient. To compensate for high-order nonlinearity of $V_{be}$, resistor R9 is connected in series with the output terminal and fed back to the base of two transistors Q14/Q16. Due to the positive temperature dependence of resistors, by superimposing the parabolic curve of first-order compensation with the positive temperature curve of the resistor, and adjusting the value of resistor R9, a reference of high-order compensation can be obtained.

## 3. Post Simulation Results

**Figure 10:**
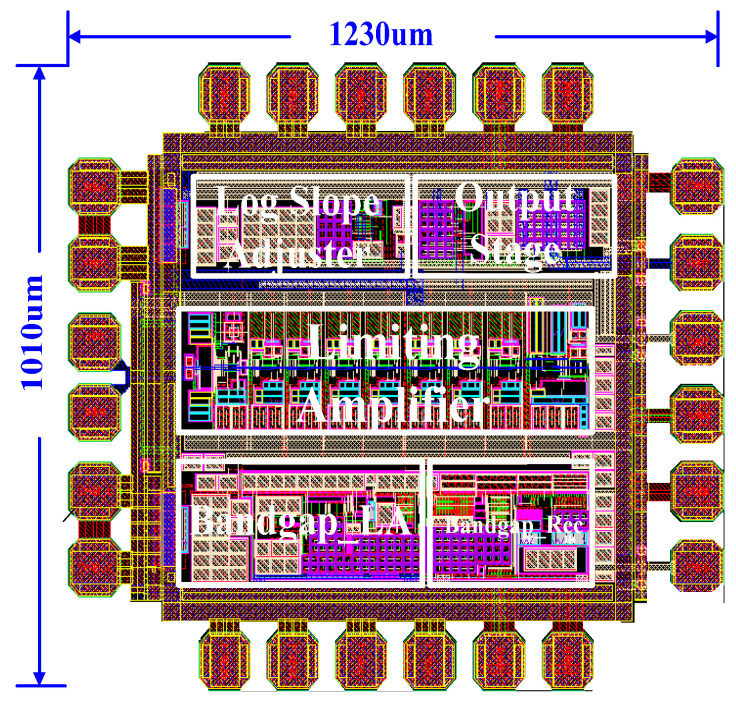
Layout of logarithmic amplifier.

The logarithmic amplifier is realized using 180 nm 1P6M SiGe BiCMOS technology. The power supply voltage is 5 V, and the power consumption during normal operation is 109 mW. The power consumption in power-down mode is reduced to 162 μW. The layout is shown in Figure 10, with an area of 1230 μm × 1010 μm.

**Figure 11:**
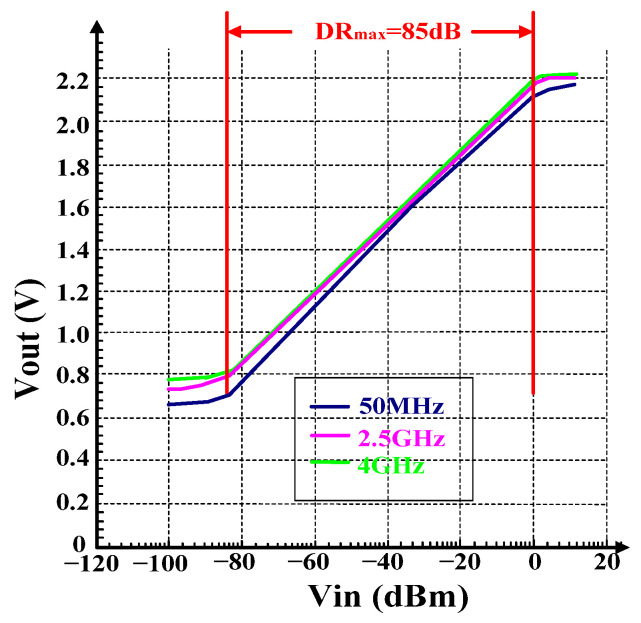
Output versus Vin.

**Figure 12:**
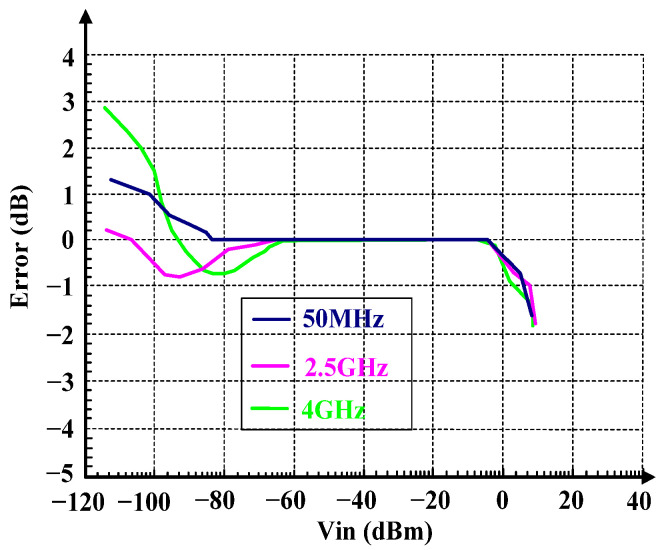
Log Conformance versus Vin.

As shown in Figure 11, under the temperature of 25 °C, when the input frequency is 50 MHz, 2.5 GHz, and 4 GHz, the dynamic ranges are 85 dB, 83 dB, and 82 dB, respectively. The log slopes are 16.8 mV/dB, 16.9 mV/dB, and 17.1 mV/dB, respectively, and the minimum detectable input power is −85 dBm, which is also the noise floor. Within the dynamic range, the log error range is as shown in Figure 12, and the overall range is within ±1 dB.

**Figure 13:**
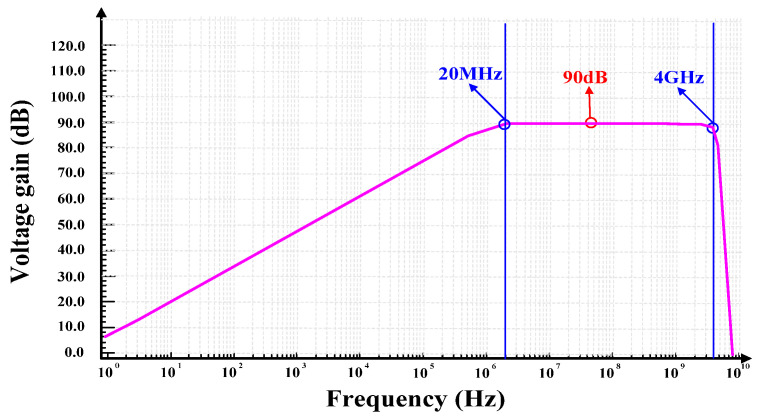
AC response.

The AC response of the logarithmic amplifier is shown in Figure 13. With a power supply voltage of 5 V, the 3 dB bandwidth is 20 MHz–4 GHz, and the overall gain reaches 90 dB.

**Figure 14:**
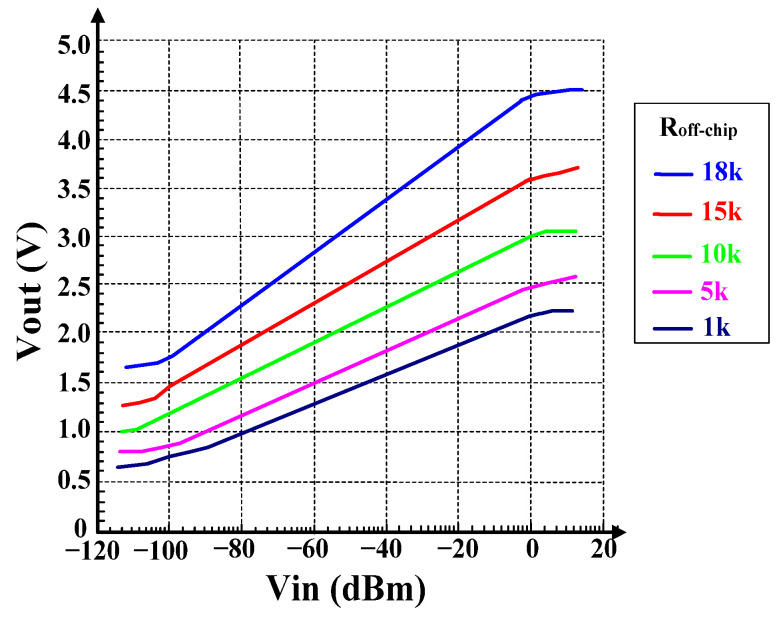
Vout Versus R_off-chip_.

Figure 14 shows the logarithmic output results of adjusting R_off-chip_ at different values. The value varies within the range of 1 kΩ–18 kΩ, and the log slope range is 16.8 mV/dB to 30.1 mV/dB. The output voltage also increases with the increase of resistance.

**Figure 15:**
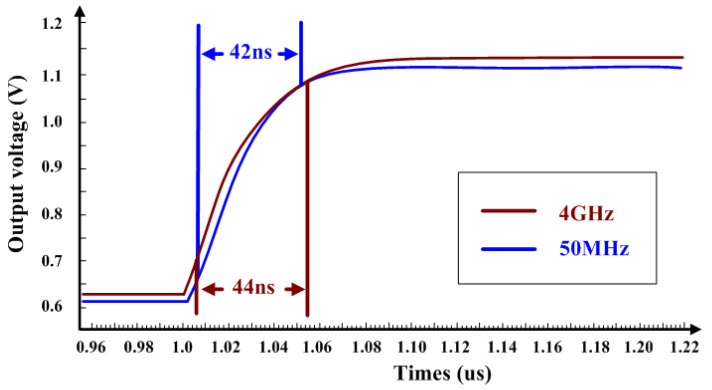
Response Time of 50 MHz and 4 GHz.

When the input frequencies are 50 MHz and 4 GHz respectively, the output is as shown in Figure 15. The response times for the output to rise from 10% to 90% amplitude are 42 ns and 44 ns, respectively, which means it has fast response to sudden input.

A comparison results of this work with others is shown in Table 1. Due to the use of the nine-stage limiting amplifier with 10 dB-gain each, a largest dynamic range has been achieved. Meanwhile, the sum of current of each stage improves the response time. Compared with previous achievements in parameters such as bandwidth, log error, and response time, it has realized a good compromise. At the same time, with a power supply of 5 V, our design also has a log slope adjuster and a power-down mode, which has more completed functions than other works. Because more limiting amplifiers are cascaded, a portion of power consumption is sacrificed to achieve performance improvement. The figure of merit (FoM) can be expressed as the ratio of power consumption to dynamic range and bandwidth, i.e., mW/(dBm × GHz). The smaller the FoM, the better the performance.

## 4. Conclusions

Based on the GSMC 180 nm SiGe BiCMOS 1P6M process, a high dynamic range and fast response logarithmic amplifier is presented. In order to meet the detection requirements of different input power, a log slope adjuster is proposed, which can adjust the slope of the logarithmic output by adjusting the off-chip resistor. Meanwhile a power-down function is also added, which can significantly reduce power consumption in standby mode. The post-simulation results show that at a power supply voltage of 5 V, 3 dB bandwidth of 4 GHz is achieved, while the logarithmic error is only ±1 dB. The dynamic range can reach over 80 dB with a response time of 42 ns. Meanwhile the adjustable log slope range is 16.8–30.1 mV/dB. Under normal operation and power-down modes, the power consumption is 109 mW and 162 μW, respectively.

## Figures and Tables

**Table 1:** Performance comparison.

Parameter	[10]	[12]	[14]	[22]	[23]	This Work
Process	180 nm CMOS	350 nm CMOS	130 nm CMOS	180 nm CMOS	180 nm CMOS	180 nm SiGe BiCMOS
Power supply (V)	3.3	2.5	2	1.8	1.8	5
Bandwidth (GHz)	0.001	2.4	16	1.8	10.6	4
Dynamic range (dB)	70	43	43	29	20	85
Log error (dB)	N/A *	N/A	±1.5	±1	±2.4	±1
Response Time (ns)	N/A	N/A	N/A	N/A	N/A	42
log slope adjustment	N/A	N/A	No	No	No	Yes
Pdc (mW)	19	184	35.2	16	10.8	70
FoM (mW/(dBm × GHz))	271.4	1.78	0.051	0.31	0.051	0.21

* “N/A” means the parameter is not listed in the paper.

## Data Availability

All the data are reported/cited in the paper.
